# Performance and microbial characteristics of biomass in a full-scale aerobic granular sludge wastewater treatment plant

**DOI:** 10.1007/s11356-017-0615-9

**Published:** 2017-11-03

**Authors:** Piotr Świątczak, Agnieszka Cydzik-Kwiatkowska

**Affiliations:** Department of Environmental Biotechnology, University of Warmia and Mazury in Olsztyn, Sloneczna 45G, 10-709 Olsztyn, Poland

**Keywords:** Granular sludge, Metagenome, Next-generation sequencing (NGS), WWTP, Sludge management

## Abstract

By modification of the operational conditions of batch reactors, a municipal wastewater treatment plant was upgraded from activated sludge to aerobic granular sludge (AGS) technology. After upgrading, the volume of the biological reactors was reduced by 30%, but the quality of the effluent substantially improved. The concentration of biomass in the reactors increased twofold; the average biomass yield was 0.6 g MLVSS/g COD, and excess granular sludge was efficiently stabilized in aerobic conditions. Canonical correspondence analysis based on the results of next-generation sequencing showed that the time of adaptation significantly influenced the microbial composition of the granules. In mature granules, the abundance of ammonium-oxidizing bacteria was very low, while the abundance of the nitrite-oxidizing bacteria *Nitrospira* sp. was 0.5 ± 0.1%. The core genera were *Tetrasphaera*, *Sphingopyxis*, *Dechloromonas*, *Flavobacterium*, and *Ohtaekwangia*. Bacteria belonging to these genera produce extracellular polymeric substances, which stabilize granule structure and accumulate phosphorus. The results of this study will be useful for designers of AGS wastewater treatment plants, and molecular data given here provide insight into the ecology of mature aerobic granules from a full-scale facility.

**Electronic supplementary material:**

The online version of this article (10.1007/s11356-017-0615-9) contains supplementary material, which is available to authorized users.

## Introduction

Aerobic granular sludge (AGS) is one of the most intensively developed biological methods of wastewater treatment. In the process of biogranulation, self-immobilization of microorganisms takes place. The settling properties of granular sludge are better than those of conventional activated sludge, and granular sludge is more resistant to changes in environmental conditions, such as temperature, organic load, or pH.

The formation of aerobic granules and the morphology of the resulting granules are influenced by many operational factors such as the applied shear stress, settling time of biomass, organic loading rate, volumetric exchange rate, or hydraulic retention time (Liu and Tay 2004). Granulation is strongly influenced by the microbial composition of the biomass. Filamentous bacteria form the basis for the creation of agglomerates upon which granulation begins. These bacteria may disappear or remain in the biomass in the later stages of granulation depending on the type of available carbon source, and their presence affects granule compactness Liu and Liu (2006). The formation of strong and stable aerobic granules is favored by the presence in the biomass of bacteria that produce extracellular polymeric substances (EPSs) and slow-growing microorganisms that stabilize granule structure. The occurrence of slow-growing microorganisms can be stimulated by the presence of an anaerobic feast/aerobic famine period in the cycle of the batch reactor (Gao et al. 2011). An anaerobic fill phase promotes substrate uptake and diffusion throughout the whole granule. During this period, storage polymers are formed and slow-growing organisms predominate in the granules (Pronk et al. 2015a).

The operation of full-scale facilities with AGS ensured very efficient removal of pollutants from high-strength municipal wastewater (Gademan et al. 2010) or a mixture of 30% domestic and 70% industrial wastewater (printing, paint, textile, chemistry, and beverage industry) (Li et al. 2014). AGS allows the dimensions of a wastewater treatment plant (WWTP) to be reduced. In the Frielas WWTP (Portugal), modification of 9% of the total biological volume of the plant to use aerobic granular sludge technology allowed this part of the plant to treat 25% of the total flow and produced better quality effluent than the activated sludge reactors (https://www.royalhaskoningdhv.com). The use of aerobic granules also diminishes energy consumption. According to Pronk et al. (2015b), the energy use in a full-scale WWTP with aerobic granules treating domestic wastewater was 58–68% lower than in a typical conventional activated sludge plant. Despite these reports, little is known about aerobic granular sludge production and morphology in full-scale facilities. This information is crucial for designing technology for utilization of excess granular sludge.

Wastewater treatment efficiency and stability are related to the microbial structure of the biomass. Reports on aerobic granulation in WWTPs have mainly focused on technological research, and little is known about the microbial bases of wastewater purification in full-scale facilities with granular sludge. Li et al. (2014), based on the results of denaturant gradient gel electrophoresis, reported that, in a municipal WWTP with mature aerobic granules, *Flavobacterium* sp., *Aquabacterium* sp., and *Thauera* sp. occurred in granules, and *Flavobacterium* sp. supported granule formation by EPS production. Pronk et al. (2015b) investigated the microbial structure of aerobic granules from a full-scale WWTP using fluorescent in situ hybridization and observed a significant population of phosphate-accumulating organisms (PAOs), while glycogen-accumulating microorganisms were present in a low number; other heterotrophic and autotrophic bacteria were not investigated.

In recent years, molecular techniques for investigating the biomass in wastewater systems have been intensively developed, which has allowed the identification of key players in such processes as nitrification, denitrification, or phosphorus removal. A true breakthrough has been the use of next-generation sequencing (NGS, high-throughput sequencing). NGS is now regarded as the most reliable and cost-effective method for analyzing the species composition of environmental samples (Vanwonterghem et al. 2014). NGS has been successfully used for comparisons of activated sludge systems all over the world, providing information on core microbial communities and the microbial species mainly responsible for operational problems such as foaming or bulking (Zhang et al. 2012).

AGS is receiving more and more attention worldwide, but there is little information on attempts to upgrade existing full-scale WWTPs from activated sludge to aerobic granular sludge technology. To meet this need, here we report on the process efficiency and on the microbiological and morphological structure of aerobic granules during long-term operation of a municipal WWTP that was upgraded from activated sludge to aerobic granular technology by modifying the technological line and operational parameters. NGS was used to identify the microorganisms in the biomass. The results of this study will be useful for designers and operators of wastewater treatment plants with aerobic granular sludge, and the combination of technological and molecular data given here provide insight into the ecology of a mature biocenosis in aerobic granular sludge.

## Materials and methods

### Characteristics of the facility

**Fig. 1:**
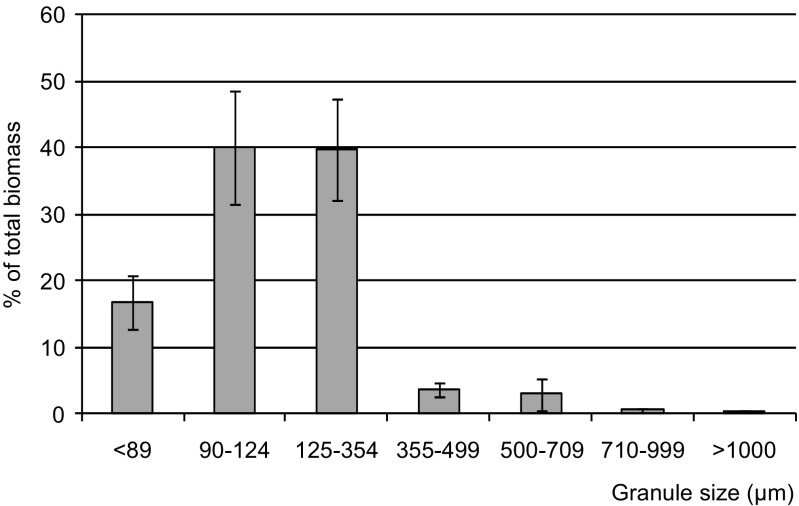
Percentage of granules with different diameters (mean of two measurements and standard deviation)

The samples were taken from the aeration tanks of the WWTP in Lubawa (Poland). This plant operates with a low organic load and now uses aerobic granular sludge technology to treat 15,000 PE (population equivalent) of wastewater. The average wastewater flow is about 3200 m^3^/day; about 30–40% of the influent was wastewater from dairy industry. The volumetric loading rate of the WWTP is 0.77 m^3^/(m^3^ day). The technological line of the wastewater treatment plant is relatively simple. First, raw sewage flows through a stepped grid, which retains floating contaminants. Then, the wastewater is pumped into a grit chamber followed by a flow equalization tank. Before upgrading, the wastewater was pumped from the flow equalization tank into three biological rectangular reactors; after upgrading, the wastewater is pumped into two biological reactors (R1 and R2, height 4 m, working height 3 m, working volume 2080 m^3^, each), and the third reactor is used as an aerobic stabilization tank (Fig. 1SM). Treated wastewater from biological reactors flows to a tank equipped with a stirrer, where phosphorus precipitation can be performed if necessary. From this tank, treated wastewater is pumped to three secondary clarifiers, from which the effluent is discharged into a river. The excess sludge from the reactors is pumped to the aerobic stabilization chamber. The stabilized sludge and the sludge from the secondary clarifiers are collected in a sludge thickener. The thickened sludge is dewatered using a mechanical belt thickener (dewatering is supported by addition of polymer FLOPAM EM 840 MEB, Korona) and then composted. After upgrading, the method of wastewater feeding/discharge was changed. Before upgrading, the reactor cycle had separate phases for reactor feeding and discharge of treated wastewater. After upgrading, the cycle begins with simultaneous feeding of raw wastewater and withdrawal of effluent. The influent is pumped from the pipes at the bottom of one of the shorter sides of the rectangular biological reactor, and the effluent is collected by an overflow channel at the opposite side of the reactor. The operational mode of the reactors was modified to favor granulation (shortened settling, shorter filling, precise removal of excess sludge from aeration tanks). The whole reactor cycle lasted for 4.8 h, with an aeration period of 3.6 h. After about 2.5 h of aeration, the dissolved oxygen reaches 2 mg/L and is kept at this level until the end of the cycle. Settling lasts for 20 min, and feeding the reactor/the effluent discharge lasts for 40 min. The volumetric exchange rate was about 25%. The superficial gas velocity in the reactors was 0.18 cm/s; the sludge retention time (SRT) was about 30 days; hydraulic retention time was about 1 day, and the minimum settling velocity in the aerobic granular sludge reactors was about 1.6 m/h. In the aerobic stabilization tank, the biomass concentration reached 15–16 g mixed liquor suspended solids (MLSS)/L, and the hydraulic retention time was 17 days.

### Technological studies

The study was carried out during 11 months (the plant was upgraded in October 2015). Samples were taken once a week or once every other week. During the study, the following indicators were measured in the influent and in the effluent using cuvette tests (HACH): chemical oxygen demand (COD), total nitrogen (TN), ammonium nitrogen, and total phosphorus (TP). A DR 2800 spectrophotometer (HACH) was used for measurements. Additionally, nitrate nitrogen and nitrite nitrogen were measured in the effluent as well as nitrogen content in the biomass (APHA 1992). BOD_5_ was measured using the OxiTop system (WTW). The COD/N/P ratio in the influent was 100:7:1.5, and the volumetric organic loading rate was about 2.0 kg COD/(m^3^ day). The concentrations of TSS in the influent and of MLSS and mixed liquor volatile suspended solids (MLVSS) in the reactors and in the effluent were determined according to APHA (1992). The size of granules was estimated 6 and 7 months after the start of upgrading by a wet sieving procedure as described in Cydzik-Kwiatkowska et al. (2009). The sludge volumetric index (SVI) was measured after 5 (SVI_5_) and 30 min (SVI_30_) of settling (APHA 1992). Microscopic observations of biomass were carried out in an ECLIPSE 50i microscope (Nikon). Changes in dissolved oxygen concentration in the reactor were measured using a ProOdo probe (YSI Environmental). At the end of the study, pollutant concentrations were also measured in duplicate during the reactor cycle to examine the kinetics of removal of organics, nitrogen, and phosphorus by the aerobic granular sludge. The sludge yield coefficient (*Y*) was calculated according to Klimiuk and Kulikowska (2005). To calculate nitrification efficiency, the concentration of the oxidized nitrogen forms was divided by the concentration of TKN less the N used for biomass synthesis. For denitrification efficiency, the concentration of N reduced was divided by the concentration of all oxidized nitrogen forms. The average temperatures during the study period were 23 °C (August), 20 °C (September), 16 °C (October), 12 °C (November), 10 °C (December), 8 °C (January), 9 °C (February), and 11 °C (March).

### Microbiological analysis of granular sludge

The composition of protozoa was analyzed using an Eclipse 50i optical microscope (Nikon), and identification was based on Fiałkowska et al. (2010). Analyses were performed twice a month.

For molecular analyses, sludge from the two biological reactors was analyzed four times over 7 months. The first sample was activated sludge used as the inoculum when starting the granulation process. The next samples were taken from both reactors after 4, 6, and 7 months of operation. The collected samples were stored at − 20 °C. After thawing, DNA was isolated from the samples using a FastDNA® SPIN kit for soil (MP Biomedicals). The purity and concentration of the isolated DNA was measured using a NanoDrop solid spectrometer (Thermo Scientific). The 939F/1492 primer set (5′-TTGACGGGGGCCCGCAC-3′/5′-TACCTTGTTACGACTT-3′) was used to amplify the V6 and V8 regions of the bacterial 16S rDNA gene. The amplicons were sequenced using the Illumina MiSeq platform at Research and Testing Laboratory (USA). Depending on the sample, from 8342 to 36,496 sequences were obtained. The sequences were analyzed bioinformatically as described in Świątczak et al. (2017). To characterize microbial diversity on the level of genera, the Shannon-Wiener (*H*′) index of diversity was calculated (Hill 1973).

### Statistics

The results were statistically analyzed using Statistica 12 (StatSoft). The normality of data distribution was examined using the Shapiro-Wilk test, and the significance of differences between the results before and after the upgrading of wastewater treatment was analyzed using Student’s *t* test. The results of all statistical analyses were considered significant at *p* < 0.05. To investigate the effect of the adaptation time, temperature, and sampling point (reactor 1 or 2) on the microbial structure of biomass, canonical correspondence analysis (CCA) using Canoco 5.0 (Microcomputer Power) was used. The results were tested by unrestricted Monte Carlo permutations (499 runs).

## Results and discussion

Shifting from conventional activated sludge to aerobic granular sludge technology was cost-effective because the existing reactors were adapted for granule cultivation. Despite reducing the volume of biological reactors by about 30%, the quality of the effluent improved (Table 1) and dense, spherical granules were obtained in the reactors, which were inhabited by microbial consortia able to efficiently remove carbon, nitrogen, and phosphorus in a single reactor.

**Table 1:** Quality of influent and effluent and efficiencies of pollutant removal in the full-scale WWTP during the experimental period

Parameter	Influent (mg/L)	Before upgrading	After upgrading
		Effluent (mg/L)	
COD	1319.5 ± 235.0	119.5 ± 16.0	39.1 ± 8.2*
BOD_5_	1120 ± 260	21.0 ± 3.0	20.0 ± 2.1
TP	19.5 ± 2.1	2.7 ± 0.5	0.9 ± 0.3*
TN	90.5 ± 6.6	19.2 ± 1.9	11.8 ± 0.7*
N-NH_4_^+^	64.3 ± 4.7	10.2 ± 4.7	0.4 ± 0.5*
N-NO_3_^−^	–	3.9 ± 0.8	1.6 ± 0.6*
N-NO_2_^−^	–	0.6 ± 0.2	0.5 ± 0.2
Efficiency of COD removal	–	75.1 ± 3.8	92.3 ± 2.2*
Efficiency of TP removal	–	87.0 ± 1.9	95.1 ± 1.9*
Efficiency of nitrification	–	45.0 ± 4.7	53.2 ± 6.1*
Efficiency of denitrification	–	63.9 ± 5.1	81.7 ± 5.0*
Efficiency of TN removal	–	77.9 ± 1.7	87.2 ± 1.4*
		Reactor	
Biomass concentration	–	5222 ± 1260	9128 ± 1988

^a^Significant difference, *p* < 0.05

A high volumetric exchange rate in the system is often regarded as one of the most important factors for granulation. Lab-scale experiments at high exchange ratios (60–80%) ensured that granules predominated in the biomass, while at the smaller exchange ratios of 40 and 20%, the biomass was a mixture of aerobic granules and suspended sludge (Zhu and Wilderer 2003; Liu et al. 2005). In reports on full-scale facilities operated with AGS technology, a volumetric exchange rate of about 40–70% was used (Li et al. 2014; Pronk et al. 2015b). In the present study, the volumetric exchange ratio was only about 25%, but still, the granulation was successful. A change in the method of reactor feeding, a shorter settling time (about 1.5 h before upgrading, about 20 min after), and maintenance of a long sludge age (over 30 days) were enough to ensure that granules predominated in the biomass.

Integrating microbial ecology in the design and operation of WWTPs allows better prediction of community assembly and variations in community function in response to environmental changes. The microbial structure of the biomass was investigated during the 7 months from the start of upgrading. The first sample was a mixture of activated sludge from both aeration tanks that was used as an inoculum for granulation. The next samples for NGS were taken from both reactors when the biomass had already granulated. The diversity of biomass increased with granule maturation. The inoculum was characterized by an *H*′ of 3.50, and this index had increased to 3.93 in mature granules sampled at the end of the study.

### Biomass characteristics

One of the most important indicators of the course of granulation is the SVI. Biomass is considered granular if the SVI is below 50 cm^3^/g MLSS (Liu and Tay 2004). In this study, the values of the SVI_5_ and SVI_30_ were about 123 and 70 cm^3^/g MLSS before upgrading and about 64 and 48 cm^3^/g MLSS after upgrading, which is similar to values from other full-scale facilities with aerobic granules. For example, in a WWTP in Garmerwolde, Netherlands, the SVI_5_ and SVI_30_ were 145 and 70 cm^3^/g MLSS before upgrading and lowered to 90 and 50 cm^3^/g MLSS after upgrading, respectively (Pronk et al. 2015b). In our study, the SVI_5_/SVI_30_ ratio was about 1.76 before upgrading and 1.33 after, indicating very good compactness of the sludge bed.

The diameters of granules influence both the metabolic processes in the reactor and the settling properties of biomass. An aerobic granule has a layered structure, and the individual layers are inhabited by various species of microorganisms responsible for certain biochemical conversions. In the core of large granules, anoxic or anaerobic conditions may occur, and gases can accumulate due to microbial metabolism. In the biomass from the investigated full-scale installation, granules with sizes from 90 to 354 μm predominated, constituting about 80% of the biomass (Fig. 1). For comparison, in a full-scale WWTP treating municipal wastewater, about 80% of biomass consisted of granules larger than 0.2 mm, and more than 60% were larger than 1 mm (Pronk et al. 2015b). In granules with low diameters, such as those in the WWTP investigated in the present studies, the volume of the aerobic layer in relation to the total granule volume is the highest, which supports the growth of aerobic microorganisms such as nitrifiers or aerobic heterotrophic bacteria.

**Fig. 2:**
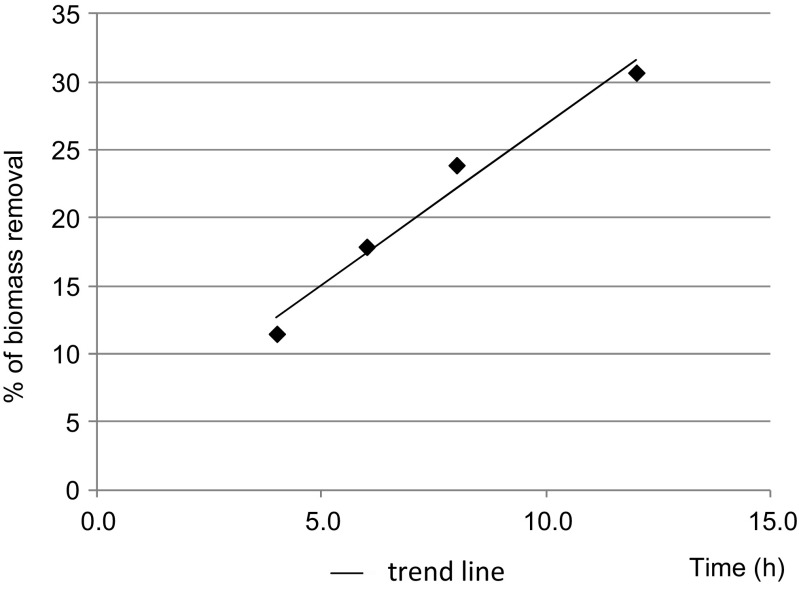
Percent of biomass removed depending on the time of stabilization

Previous reports on full-scale WWTPs with aerobic granular sludge have not reported data on aerobic granular sludge production and management. In the investigated WWTP, the sludge yield was about 0.85 g MLSS/g BOD_5_ (0.68 g MLVSS/g BOD_5_) and the food-to-microorganism ratio in the influent was about 0.09. The system was operated without a primary clarifier, and the value of sludge yield was similar to values observed in activated sludge systems. Analysis of 16 WWTPs showed that, with an average *F*/*M* value of 0.08, the average sludge production in activated sludge systems without primary clarifier was 0.86 g MLSS/g BOD_5_ (Schultz et al. 1982). Within the range investigated in the present study, the decrease in sludge concentration as a result of endogenous respiration and biomass lysis during aerobic stabilization depended on the length of aeration (Fig. 2). Every hour of aeration caused the mass of stabilized sludge to decrease by about 2.3% in comparison to the mass at the beginning of stabilization. Before stabilization, the MLVSS constituted 79% of MLSS, and after stabilization, it decreased to 60% of MLSS. Biomass from the aeration tank and from the secondary clarifiers was collected in the thickener. Due to the excellent settling properties of the aerobic granular sludge, the final concentration of biomass in this thickener reached as much as 30 g MLSS/L, and the dry weight of the biomass was 3.36%. The amount of thickened sludge for mechanical dewatering was about 55 m^3^/day. The volume of dewatered sludge produced per day was about 17 m^3^, resulting in a coefficient of dewatered sludge production of 0.005 m^3^/m^3^ of treated wastewater. The dry mass of dewatered sludge was 15–16%, which was 2–3% higher than the dry mass of dewatered activated sludge before upgrading, which allowed the overall volume of sludge generated in the WWTP to be substantially reduced.

### Treatment efficiency and microbial structure of granules

The average pollutant concentrations in the influent are presented in Table 1. Organic concentrations in the influent in the experimental period varied in a wide range from 1058.0 to 1998.0 mg COD/L. The highest COD concentrations were connected with the periods of intensive dairy production in a dairy plant near the WWTP. Ammonium nitrogen constituted about 80% of TN.

**Fig. 3:**
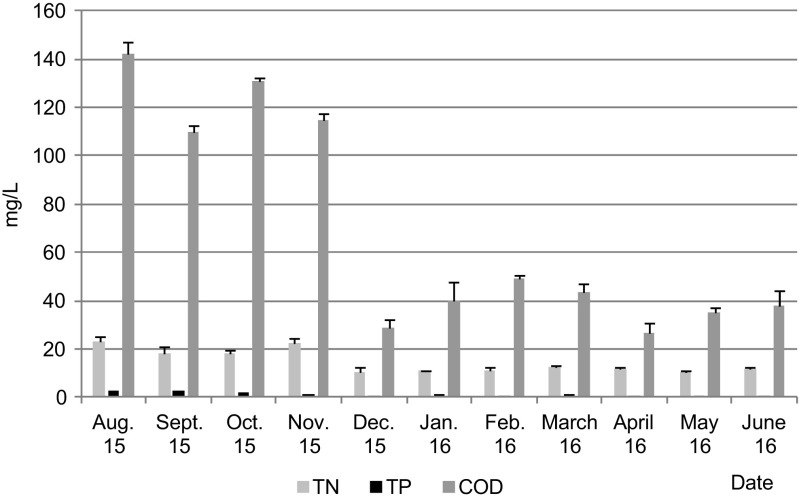
The quality of the effluent in the successive months of the experiment (upgrading started in October 2015)

**Fig. 4:**
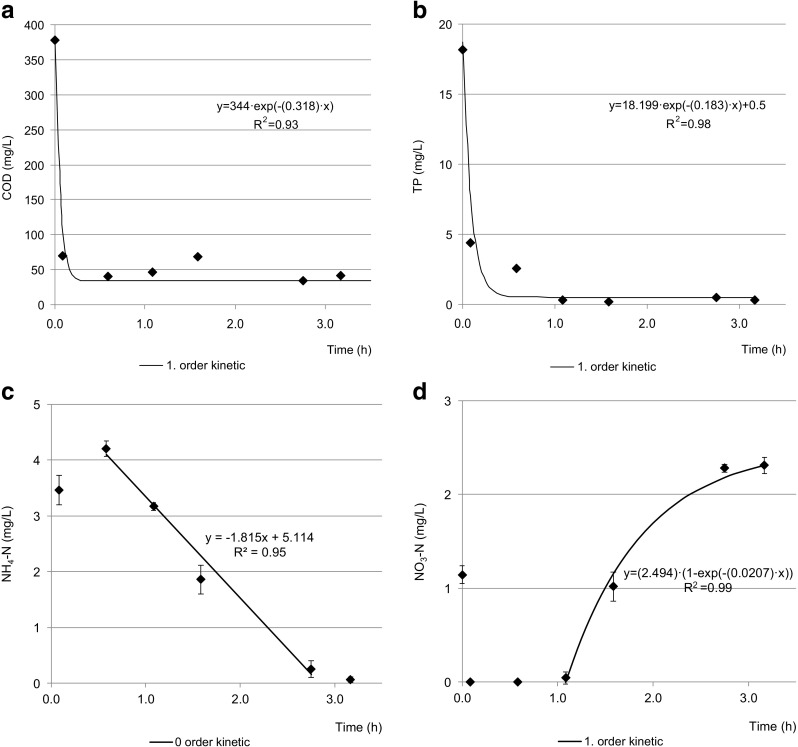
Kinetics of removal of COD (**a**), TP (**b**), ammonium nitrogen (**c**), and nitrate nitrogen (**d**); the changes in ammonium, nitrite, and nitrate nitrogen concentrations followed zero-order kinetics, while COD and TP changes followed pseudo-first and second-order kinetics, respectively

The conversion of the WWTP from activated sludge to aerobic granular sludge technology substantially improved the quality of the effluent with regard to the concentrations of COD, TP, TN, ammonium nitrogen, and nitrate (Fig. 3, Table 1). The effluent requirements were met during both summer and winter seasons; there was no need to chemically precipitate phosphorus. The average COD concentration in the effluent decreased by 67.4% after upgrading of the WWTP. The reaction rate for COD removal was 109.5 mg COD/(L h). During the first 45 min of aeration, COD decreased from about 380 to about 40 mg COD/L, indicating that almost all COD were removed in less than 1 h of the cycle (Fig. 4a). Such a quick removal of easily biodegradable substrates in the reactor due to storage of polymers in microbial cells or absorption of organics on granules in the feeding phase favors stable granulation (Pronk et al. 2015a).

During the first 30 min of aeration, ammonium concentration increased due to ammonification; then, ammonium nitrogen removal proceeded in a linear manner (Fig. 4c) with a removal rate of 1.82 mg/(L h). All ammonium was removed from wastewater after 3 h of aeration. The kinetics of nitrite removal is not presented here because its concentration did not exceed 0.6 mg/L during the aeration phase. At the beginning of aeration, the concentration of nitrates left over from the previous cycle was about 1.5 mg/L, but this was quickly reduced to 0. After about 1 h of aeration, the nitrate concentration started to gradually increase at a rate of 2.49 mg/(L h) and reached 2.25 mg/L after 3.5 h of aeration (Fig. 4d).

The overall abundance of ammonium-oxidizing bacteria (AOB) was very low (about 0.1%) with *Nitrosomonas* and *Nitrosovibrio* as the most numerous genera. These results are in agreement with those from activated sludge systems. In full-scale WWTPs with activated sludge, *Nitrosomonas* and *Nitrosospira* accounted for 0–0.34 and 0–0.20% of bacteria, respectively (Wang et al. 2014), and AOB made up 0–0.64% of bacteria (Zhang et al. 2011a). Despite these low abundances, very high efficiency of ammonium removal was observed in both studies. In our study, ammonium oxidation by the aerobic granules was better than that in the activated sludge because, despite the low percentage of AOB in the granules, the concentrations of biomass in the reactor increased significantly from 5222 ± 1260 mg MLSS/L at the beginning of the study to 9128 ± 1988 mg MLSS/L after 7 months of reactor operation (Table 1). The only nitrite-oxidizing bacteria (NOB) identified in the granules belonged to *Nitrospira* sp. They constituted about 1.6% of the inoculum, and their abundance decreased substantially (*R* = − 0.98) to about 0.5% in mature granules (Table 2). This decrease may have resulted from changes in the biomass structure. There are no diffusion limits in activated sludge, whereas oxygen penetration into the granules is more restricted. NOB have a lower affinity for oxygen than, for example, AOB (Bin et al. 2011), so conditions for NOB growth in activated sludge were better than in granular sludge.

**Table 2:** Percentage in samples of the most important classes, families, genera, and species

Class (phylum)^a^	Family (order)^a^	Genus^a^	Species^a^	Inoc.	4^b^	6^b^	7^b^
Actinobacteria (Actinobacteria)				32.7	31.2 ± 4.1	32.1 ± 2.6	14.3 ± 3.7
	Intrasporangiaceae (Actinomycetales)			12.8	6.7 ± 6.7	3.8 ± 0.2	6.7 ± 2.9
		*Tetrasphaera*		12.8	6.7 ± 3.8	3.8 ± 0.2	6.7 ± 2.9
			*Tetrasphaera jenkinsii*	2.9	0.7 ± 1.0	0.0	0.3 ± 0.0
			*Tetrasphaera australiensis*	0.0	0.6 ± 0.8	0.6 ± 0.2	0.0
	Streptomycetaceae (Actinomycetales)			0.2	0.3 ± 0.3	0.7 ± 0.0	0.0
		*Streptomyces*		0.2	0.3 ± 0.3	0.7 ± 0.0	0.0
	Acidothermaceae (Acidothermales)			1.2	1.0 ± 0.4	0.4 ± 0.1	0.0
		*Acidothermus*		1.2	1.0 ± 0.4	0.4 ± 0.0	0.0
			*Acidothermus cellulolyticus*	1.2	1.0 ± 0.4	0.4 ± 0.0	0.0
	Mycobacteriaceae (Corynebacteriales)			0.3	0.2 ± 0.3	0.5 ± 0.0	0.3 ± 0.0
		*Mycobacterium*		0.3	0.4 ± 0.0	0.5 ± 0.0	0.3 ± 0.0
Acidimicrobiia (Actinobacteria)				0.7	0.5 ± 0.3	0.0	0.1 ± 0.0
Gammaproteobacteria (Proteobacteria)				9.5	15.6 ± 1.2	14.7 ± 0.8	9.7 ± 0.4
	Xanthomonadaceae (Xanthomonadales)			0.6	3.4 ± 3.5	3.9 ± 0.2	2.6 ± 0.2
		*Thermomonas*		0.3	0.6 ± 0.3	0.4 ± 0.0	0.6 ± 0.2
		*Arenimonas*		0.1	0.1 ± 0.0	0.6 ± 0.2	1.8 ± 0.1
	Rhodanobacteraceae (Xanthomonadales)			1.5	3.0 ± 4.3	3.7 ± 0.8	1.9 ± 0.3
		*Dokdonella*		0.8	4.9 ± 0.4	2.0 ± 0.2	0.8 ± 0.0
		*Dyella*		0.5	0.7 ± 0.2	0.7 ± 0.1	0.9 ± 0.2
			*Dyella japonica*	0.5	0.6 ± 0.2	0.6 ± 0.1	0.9 ± 0.2
		*Dongia*		0.1	0.0	0.0	0.7 ± 0.0
			*Dongia rigui*	0.1	0.0	0.0	0.7 ± 0.0
	Competibacteraceae			3.5	5.8 ± 2.6	3.7 ± 0.8	1.2 ± 0.3
		*Candidatus Competibacter*		3.5	5.8 ± 2.6	3.7 ± 0.8	1.2 ± 0.1
	Aeromonadaceae (Aeromonadales)			0.2	0.4 ± 0.1	0.7 ± 0.0	0.8 ± 0.2
		*Tolumonas*		0.2	0.4 ± 0.1	0.6 ± 0.0	0.7 ± 0.4
	Moraxellaceae (Pseudomonadales)			0.2	0.0	0.2 ± 0.1	0.7 ± 0.0
		*Alkanindiges*		0.1	0.0	0.1 ± 0.1	0.7 ± 0.0
Alphaproteobacteria (Proteobacteria)				8.3	10.6 ± 1.7	13.4 ± 1.1	13.4 ± 0.5
	Hyphomicrobiaceae (Rhizobiales)			0.9	1.7 ± 0.4	2.5 ± 0.2	1.2 ± 0.1
		*Devosia*		0.6	1.3 ± 0.3	2.1 ± 0.2	1.0 ± 0.1
	Phyllobacteriaceae (Rhizobiales)			0.7	1.0 ± 0.3	1.6 ± 0.1	0.5 ± 0.1
		*Mesorhizobium*		0.7	1.0 ± 0.3	1.6 ± 0.1	0.5 ± 0.1
	Methylocystaceae (Rhizobiales)			0.6	0.6 ± 0.1	0.8 ± 0.0	0.3 ± 0.0
		*Methylocystis*		0.4	0.5 ± 0.1	0.7 ± 0.0	0.3 ± 0.0
	Rhizobiaceae (Rhizobiales)			0.4	1.1 ± 0.5	0.8 ± 0.0	1.1 ± 0.1
	Sphingomonadaceae (Sphingomonadales)			0.7	0.8 ± 0.7	1.3 ± 0.4	4.8 ± 0.0
		*Sphingopyxis*		0.6	0.7 ± 0.7	1.2 ± 0.4	4.6 ± 0.4
	Rhodobacteraceae (Rhodobacterales)			0.5	1.0 ± 0.0	1.1 ± 0.0	0.7 ± 0.0
		*Paracoccus*		0.1	0.6 ± 0.2	0.7 ± 0.0	0.2 ± 0.0
	Rhodospirillaceae (Rhodospirillales)			0.6	0.4 ± 0.1	0.8 ± 0.2	0.9 ± 0.3
	Caulobacteraceae (Caulobacterales)			0.5	0.7 ± 0.2	0.8 ± 0.1	0.6 ± 0.1
Betaproteobacteria (Proteobacteria)				3.9	5.7 ± 0.2	5.8 ± 0.4	12.2 ± 1.7
	Rhodocyclaceae (Rhodocyclales)			0.6	1.6 ± 0.6	1.8 ± 0.1	5.9 ± 1.8
		*Dechloromonas*		0.1	0.4 ± 0.0	0.8 ± 0.0	4.2 ± 1.7
		*Zoogloea*		0.0	0.1 ± 0.1	0.1 ± 0.1	1.1 ± 0.1
	Comamonadaceae (Burkholderiales)			0.8	1.2 ± 0.3	1.0 ± 0.0	1.9 ± 0.2
		*Rhodoferax*		0.3	0.3 ± 0.4	0.2 ± 0.0	1.0 ± 0.1
	Burkholderiaceae (Burkholderiales)			0.6	0.3 ± 0.0	0.3 ± 0.2	0.0
		*Burkholderia*		0.6	0.3 ± 0.0	0.1 ± 0.1	0.0
Deltaproteobacteria (Proteobacteria)				2.9	3.6 ± 0.9	4.7 ± 0.2	9.5 ± 1.8
	Polyangiaceae (Myxococcales)			0.1	0.2 ± 0.1	0.2 ± 0.0	0.9 ± 0.5
	Kofleriaceae (Myxococcales)			0.0	0.2 ± 0.1	0.3 ± 0.0	0.8 ± 0.2
	Bdellovibrionaceae (Bdellovibrionales)			0.3	0.1 ± 0.1	0.2 ± 0.1	0.7 ± 0.3
		*Bdellovibrio*		0.3	0.1 ± 0.1	0.2 ± 0.1	0.7 ± 0.3
Sphingobacteriia (Bacteroidetes)				7.4	4.8 ± 2.0	4.2 ± 0.2	9.5 ± 2.1
	Saprospiraceae (Sphingobacteriales)			1.1	0.6 ± 0.3	0.6 ± 0.1	1.2 ± 0.1
		*Lewinella*		0.7	0.4 ± 0.1	0.4 ± 0.2	0.6 ± 0.3
Flavobacteria (Bacteroidetes)				1.3	1.8 ± 0.3	2.0 ± 0.7	4.0 ± 0.2
	Flavobacteriaceae (Flavobacteriales)			0.2	1.2 ± 0.1	1.4 ± 0.8	2.1 ± 0.1
		*Flavobacterium*		0.2	1.2 ± 0.1	1.4 ± 0.8	2.0 ± 0.6
			*Flavobacterium longum*	0.1	0.4 ± 0.0	0.8 ± 0.7	0.7 ± 0.0
Cytophagia (Bacteroidetes)				0.3	1.8 ± 0.9	2.6 ± 0.6	3.9 ± 0.8
	Cytophagaceae (Cytophagales)			0.2	1.1 ± 0.9	1.9 ± 0.4	2.3 ± 0.5
		*Ohtaekwangia*		0.2	1.1 ± 0.9	1.9 ± 0.4	2.2 ± 0.7
Acidobacteriia (Acidobacteria)				1.8	1.8 ± 0.2	1.7 ± 0.1	1.8 ± 0.2
	Acidobacteriaceae (Acidobacteriales)			1.3	1.1 ± 0.1	1.1 ± 0.0	1.2 ± 0.1
		*Acidobacterium*		1.2	1.1 ± 0.1	1.1 ± 0.0	1.1 ± 0.1
Nitrospira (Nitrospirae)				1.7	0.8 ± 0.2	0.8 ± 0.0	0.5 ± 0.1
	Nitrospiraceae (Nitrospirales)			1.6	0.8 ± 0.2	0.8 ± 0.0	0.5 ± 0.1
		*Nitrospira*		1.6	0.8 ± 0.2	0.8 ± 0.0	0.5 ± 0.1
Thermomicrobia (Chloroflexi)				0.3	0.5 ± 0.3	0.7 ± 0.1	0.2 ± 0.1
	Sphaerobacteraceae (Sphaerobacterales)			0.3	0.5 ± 0.3	0.7 ± 0.1	0.0
		*Sphaerobacter*		0.3	0.5 ± 0.3	0.7 ± 0.1	0.2 ± 0.1
Caldilineae (Chloroflexi)				1.1	0.5 ± 0.1	0.5 ± 0.0	0.3 ± 0.1
	Caldilineaceae (Caldilineales)			1.0	0.5 ± 0.1	0.4 ± 0.0	0.2 ± 0.1
		*Caldilinea*		1.0	0.5 ± 0.1	0.4 ± 0.0	0.2 ± 0.1
Anaerolineae (Chloroflexi)				0.8	0.5 ± 0.0	0.5 ± 0.0	0.2 ± 0.0
Chloroflexia (Chloroflexi)				0.4	0.1 ± 0.1	0.0	0.7 ± 0.1
Clostridia (Firmicutes)				0.8	1.0 ± 0.1	0.8 ± 0.1	0.4 ± 0.0
	Clostridiaceae (Clostridiales)			0.4	0.5 ± 0.1	0.4 ± 0.0	0.3 ± 0.0
Bacilli (Firmicutes)				0.6	0.4 ± 0.2	0.7 ± 0.2	0.3 ± 0.1
Ignavibacteria (Ignavibacteriae)				0.7	0.6 ± 0.1	0.3 ± 0.1	0.1 ± 0.0
	Ignavibacteriaceae (Ignavibacteriales)			0.7	0.6 ± 0.1	0.3 ± 0.1	0.0
		*Ignavibacterium*		0.7	0.6 ± 0.1	0.3 ± 0.1	0.1 ± 0.0
Planctomycetia (Planctomycetes)				0.6	0.4 ± 0.3	0.6 ± 0.0	0.3 ± 0.1
Verrucomicrobiae (Verrucomicrobia)				0.4	0.5 ± 0.0	1.1 ± 0.3	0.7 ± 0.1

*Inoc* inoculum

^a^No data is presented for taxa whose abundance in any sample did not cross a threshold of 0.5%

^b^Month of sampling, the average from R1 and R2

The upgrading of the WWTP significantly increased total nitrogen removal (Table 1). The average TN concentration in the effluent decreased from 19.2 ± 1.9 to 11.7 ± 0.8 mg/L due to a significant decrease in the concentrations of ammonium nitrogen and nitrates. Because granules have a more compact structure than activated sludge, denitrification efficiency increased from about 64% at the beginning of the experiment to about 82% after upgrading, despite constant aeration of the reactor during its cycle.

Denitrifiers belong to a broad range of phylogenetic groups, which makes them difficult to investigate. Their species structure in activated sludge is influenced by the type of technological system; the highest diversity of N_2_O reducers has been detected in WWTPs with separated denitrification tanks (Jaranowska et al. 2013). In most WWTPs, denitrifiers from *Thauera* sp. are usually detected (Jiang et al. 2008); in the present study, however, the percentage of *Thauera* sp. did not exceed 0.5% in any of the investigated samples. In the granules, other taxa with denitrification ability were present. For example, the abundance of Sphingobacteriia, including the family Saprospiraceae, was high throughout the study and was highest in mature granules (9.5 ± 2.1%). Members of Saprospiraceae are acetotrophic denitrifiers (Adav et al. 2010a) able to degrade hydrocarbons and proteins (Sack et al. 2014). Their high abundance in the system may have been caused by the large input of protein-rich dairy wastewater; this industrial stream constituted up to 40% of all influent. Another group of microorganisms able to oxidize organics and conduct nitrification-denitrification processes is Comamonadaceae (Li et al. 2008). Their number was highest in the mature granular sludge (1.9 ± 0.2%), although their abundance did not vary significantly during the study. Previous findings by Adav et al. (2010b) and Ginige et al. (2005) suggested that, in lab-scale aerobic granular sludge systems, the members of the Comamonadaceae family play a major role in denitrification processes in the presence of acetate.

In the present study, the average total phosphorus concentration in the effluent significantly decreased from 2.7 ± 0.5 mg/L before upgrading to 0.9 ± 0.3 mg/L afterwards, for a TP removal efficiency of 95.1 ± 1.9%. Biological phosphorus removal is most intense in anaerobic/aerobic systems. In the investigated WWTP, there is no separate anaerobic phase, but during reactor feeding, the oxygen concentration drops and anaerobic conditions are created. Efficient phosphorus removal indicates that this period (about 16% of the cycle length) was sufficient to release orthophosphate (approximately 14–15 mg/L), which was then stored in the aerobic phase. TP removal proceeded quickly during the first hour of aeration, and the reaction rate for TP removal was 5.16 mg/(L h) (Fig. 4b).

The enhanced removal of phosphorus requires the presence of heterotrophic PAOs that accumulate intracellular phosphorus in amounts higher that their natural requirements in varying anaerobic/aerobic conditions. In the investigated system, the PAO family Intrasporangiaceae (Hanada et al. 2002) was one of the core microbial communities. Their abundance was 12.8% in the inoculums, and between 3.8 ± 0.2 and 6.7 ± 2.9% in the aerobic granules. *Accumulibacter* sp. and *Tetrasphaera* sp. are usually the most abundant PAOs in full-scale WWTPs with activated sludge (Kong et al. 2005; Nguyen et al. 2011). In our studies, *Tetrasphaera* sp. comprised up to 6.7 ± 2.9% of the bacterial community in mature granules and was the most numerous genus of PAO, whereas *Accumulibacter* sp. was not observed. This observation can also be explained by the fact that large quantities of dairy proteins were present in the influent, which were a source of amino acids, a preferred substrate for *Tetrasphaera*-related PAOs (Nguyen et al. 2011). The high abundance of *Tetrasphaera*-related PAOs is favorable for the treatment process because their ecophysiology seems to be more versatile than that of *Accumulibacter* sp. *Tetrasphaera*-related PAOs take up glucose and ferment this to create succinate and other components. They synthesize glycogen as a storage polymer and use it in aerobic conditions to provide energy for growth and to replenish their intracellular polyphosphate reserves. Finally, they are also able to denitrify (Kristiansen et al. 2013). In the biomass, other denitrifying PAOs belonging to Rhodocyclaceae were also present. In this study, they were represented mostly by the genus *Dechloromonas*, whose abundance increased from 0.1% in the inoculum to 4.2 ± 1.7% in mature granules. Rhodocyclaceae occur in mature granules at temperatures ranging from 20 to 35 °C (Ebrahimi et al. 2010) and are important for formation of a layered structure of granules. They create a granule core covered by an outer spherical shell inhabited by, e.g., Flavobacteriaceae, Xanthomonadaceae, and Rhodobacteraceae (Lv et al. 2014).

**Fig. 5:**
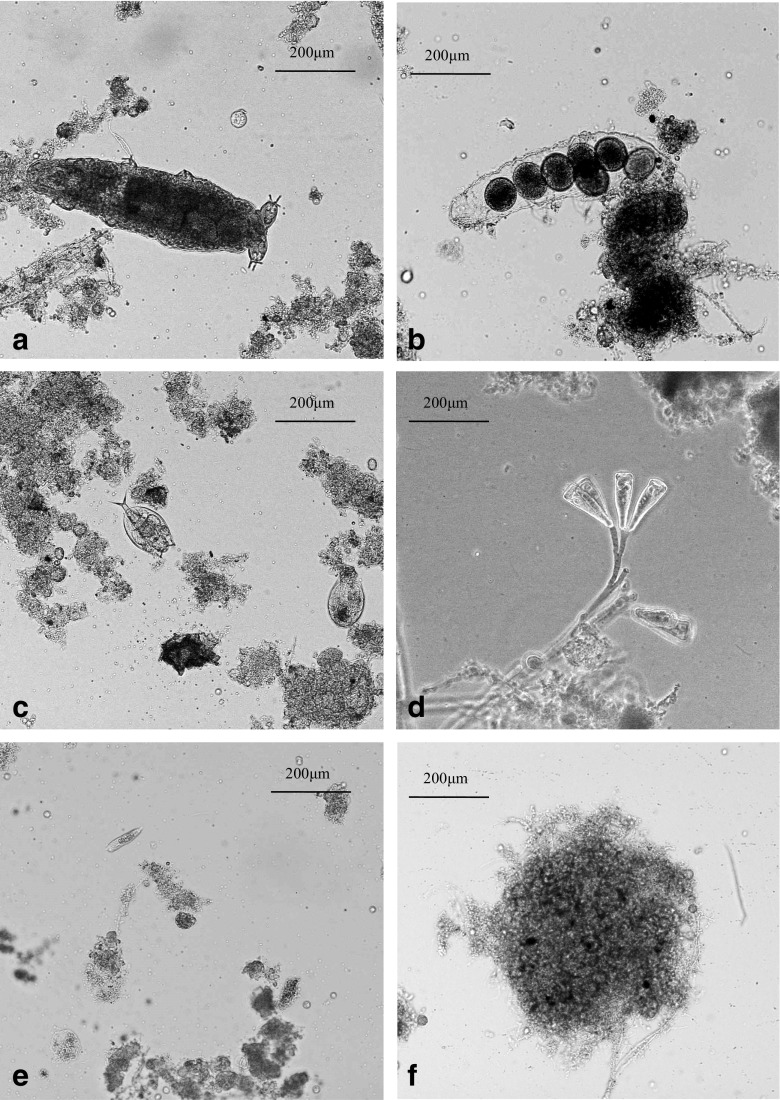
Granular sludge: tardigrade (**a**), tardigrade eggs (**b**), rotifer (**c**), *Voricella* sp. (**d**), *Aspidisca* sp. (**e**), and morphology (**f**)

Microscopic observations revealed that granules were densely packed with microorganisms. The surface of granules was inhabited by tardigrades, sedentary and walking ciliates, and rotifers (Fig. 5). The presence of these organisms indicates proper aerobic conditions in the reactors and a healthy, low-loaded microbial ecosystem. Tardigrades and rotifers feed on free-floating bacteria and small bacterial consortia. Protozoa, especially ciliates (Fig. 5d), efficiently remove small particulate matter from wastewater. In the present study, their activity resulted in very low concentrations of TSS in the effluent (about 15 mg TSS/L). In the aerobic granular sludge, walking ciliata were identified belonging to *Aspidisca* sp., which feed on dead and irregular bacterial consortia on the granule surface, causing the granules to have a clear outer shape (Fig. 5f).

### Canonical correspondence analysis

**Fig. 6:**
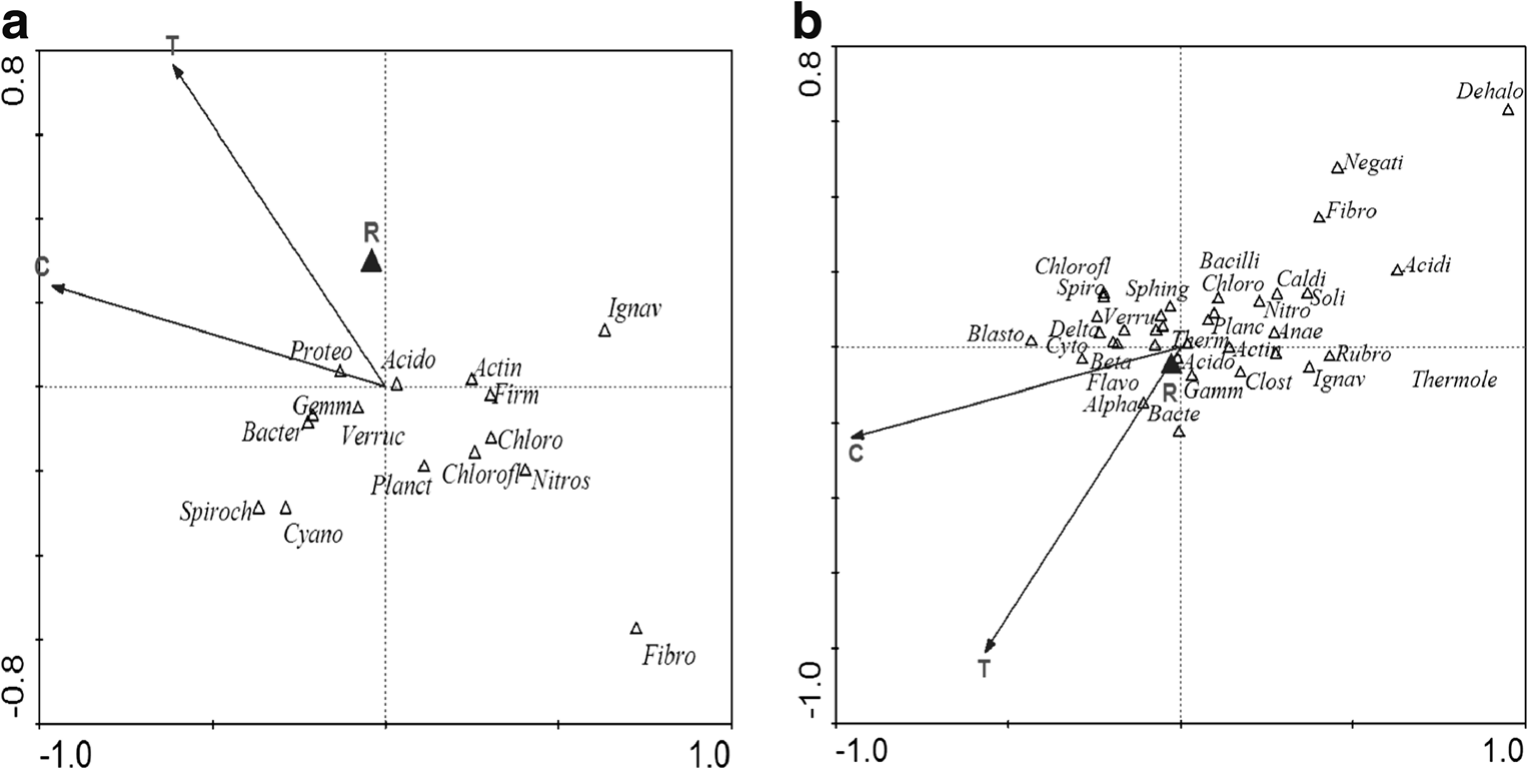
CCA showing the association between bacterial **a** phyla and operational variables; the continuous operational variables (temperature (T), the number of cycles from the start of granulation (C)) are represented by arrows; the discontinuous operational variable (reactor (R) is represented by a triangle. Actin Actinobacteria, Proteo Proteobacteria, Bacter Bacteroidetes, Chlorofl Chloroflexi, Acido Acidobacteria, Firmicut Firmicutes, Nitrospi Nitrospirae, Planctom Planctomycetes, Ignav Ignavibacteriae, Verruc Verrucomicrobia, Gemm Gemmatimonadetes, Spriroch Spirochaetes, Fibro Fibrobacteres, Chloro Chlorobi, Cyano Cyanobacteria. **b** Classes and operational variables. Acidi Acidimicrobiia, Acido Acidobacteriia, Actin Actinobacteria, Alpha Alphaproteobacteria, Anae Anaerolineae, Bacilli, Bacte Bacteroidia, Beta Betaproteobacteria, Blasto Blastocatellia, Caldi Caldilineae, Chloro Chlorobia, Chlorofl Chloroflexia, Clost Clostridia, Cyto Cytophagia, Dehalo Dehalococcoidia, Delta Deltaproteobacteria, Fibro Fibrobacteria, Flavo Flavobacteriia, Gamm Gammaproteobacteria, Ignav Ignavibacteria, Negati Negativicutes, Nitro Nitrospira, Planc Planctomycetia, Rubro Rubrobacteria, Soli Solibacteres, Sphing Sphingobacteriia, Spiro Spirochaetia, Thermole Thermoleophilia, Therm Thermomicrobia, Verru Verrucomicrobiae

To investigate the influence of temperature (*T*), the number of cycles from the start-up of the aerobic granular sludge reactor (C, time of adaptation), and the reactor (two identically operated reactors, R1 and R2) on the microbial structure of the biomass, CCA was performed at the phylum, class, family, genus, and species levels. At the phylum level, CCA showed that these environmental variables explained 92.3% of the variability in the GSBR (granular sludge batch reactor) community and that only the time of adaptation significantly influenced the microbial composition of the granules (*p* = 0.040). Actinobacteria, Nitrospirae, Chloroflexi, Chlorobi, Firmicutes, and Ignavibacteriae were more numerous in the activated sludge, while Proteobacteria, Spirochaetes, Cyanobacteria, Bacteroidetes, and Verrucomicrobia were more abundant in the granular sludge (Fig. 6a). The predominance of Proteobacteria (up to 47.2% of sequences) in this study is consistent with observations of both activated sludge (Zhang et al. 2011b) and granular sludge systems (Liao et al. 2013).

At the class level, only the time of adaptation had a significant influence on the microbial composition (*p* = 0.038), and all of the analyzed environmental variables explained 84.9% of the variability in the microbial composition of the aerobic granules. The abundances of Acidimicrobiia, Nitrospira, Caldilineae, Anaerolineae, and Ignavibacteria were higher in the activated sludge (Fig. 6b), while the abundances of Alphaproteobacteria, Betaproteobacteria, Deltaproteobacteria, Flavobacteria, and Cytophagia were greater in the mature granules. Betaproteobacteria is usually the most abundant class of bacteria in activated sludge, and the fact that their abundance in the aerobic granules at the end of the study was more than three times as much as in the activated sludge is favorable because they are largely responsible for organic and nutrient removal (Nielsen et al. 2010; Hu et al. 2012; Wang et al. 2012).

Molecular studies identified core microbial communities in the WWTP that were present in the biomass throughout the study. These communities are probably crucial for proper functioning of wastewater treatment systems, independent of the type of biomass. In the present study, the most abundant class was Actinobacteria (14.3 ± 3.7 to 32.7%). Other classes with relatively stable abundance included Gammaproteobacteria (9.5 to 15.6 ± 1.2%), Sphingobacteriia (4.2 ± 0.2 to 9.5 ± 2.1%), Acidobacteriia (about 1.8%), Clostridia (from 0.4 ± 0.0 to 1.0 ± 0.1%), Bacilli (on average 0.5%), and Planctomycetia (on average 0.5%).

The results of the present study indicate a transition in microbial structure during granule maturation from backbone-forming filamentous bacteria in activated sludge to EPS-producing bacteria in aerobic granules. The investigated WWTP encountered problems with sludge bulking and foaming before upgrading. These problems were mainly caused by variations in the organic loading and wastewater composition caused by discharges of dairy wastewater. After upgrading to granular technology, no bulking or foaming was observed. This could be connected with the changes in the microbial structure of the biomass. In most activated sludge WWTPs, the separation of biomass from treated wastewater is eventually disrupted by excessive growth of some Actinobacteria, leading to problems with bulking and foaming (Jenkins et al. 2004; Tandoi et al. 2006). Actinobacteria are slow-growing k-strategists (Rossetti et al. 2005); therefore, they are integrated into the activated sludge flocs, ensuring their retention in the system by recycling in the return activated sludge. In the present study, the abundance of Actinobacteria decreased from 32.7% in activated sludge to 14.3 ± 3.7% in mature granules at the end of the experiment. Also, the abundances of the genus *Caldilinea* had a strong negative correlation (*R* = − 0.98, *p* < 0.05) with the time of transition of biomass from activated to mature aerobic granular sludge. Among *Caldilinea* sp., there are many filamentous species that are regarded as capable of stabilization of activated sludge flocs (Yoon et al. 2010).

CCA showed that none of the environmental variables significantly influenced the microbial structure of the granules on the levels of order, family, genus, and species, although clear trends in changes in abundance were observed in particular taxa. This was especially noticeable in the case of taxa that play an important role in the formation and growth of aerobic granules and help to sustain the structure of granular sludge in bioreactors. The abundance of bacteria belonging to the order Xanthomonadales increased from 2.1% in activated sludge to 4.5–7.6% in aerobic granules. Also, the abundance of bacteria belonging to the order Sphingomonadales increased during the process of biomass adaptation and reached 4.8 ± 0.0% in the mature granules, in comparison to 0.6% in the activated sludge. Microorganisms belonging to both these orders excrete EPSs. Microorganisms belonging to Xanthomonadaceae produce *N*-acyl-homoserine-lactone, which is a component of EPS and plays an important role in quorum-sensing, which leads to bacterial aggregation (Tan et al. 2014). Members of Sphingomonadaceae constituted an important component of biomass in lab-scale reactors with aerobic granules operated at a wide range of temperatures (20–30 °C) in the period of granule maturation and in mature biomass. Sphingomonadaceae are present in nitrogen-converting systems with aerobic granules for both ammonium removal (Wan et al. 2013) and denitrification (Liao et al. 2013). Another group that can be regarded as important for granule formation is Rhizobiales. In our study, the most abundant family of Rhizobiales in granules was Hyphomicrobiaceae, accounting for 0.9% of inoculum and from 1.2 to 2.5% of granules. Hyphomicrobiaceae possess one or more sticky filaments that mediate the adherence of bacteria to surfaces (Rainey et al. 1998). *Candidatus competibacter* was the most numerous species identified in the biomass, with an abundance up to 5.8 ± 2.6% in the samples taken after 4 months of reactor operation. *C. competibacter* was demonstrated to be essential for granulation through the production of granulan associated with EPS (Seviour et al. 2011). Our studies point to their relatively stable abundance in a wastewater system independently of the type of biomass, which may indicate that they are generally important for creation of complex microbial communities in wastewater treatment systems.

There were strong correlations on the genus level between the abundances of genera *Ohtaekwangia* (*R* = 0.99) and *Flavobacterium* (*R* = 0.99) and the time of adaption; in mature granules, the abundance of these taxa reached 2.2 ± 0.7 and 2.0 ± 0.6%, respectively. There was also a strong negative correlation on the species level: the abundance of *Acidothermus cellulolyticus* (*R* = − 0.96) diminished with the transition of biomass from activated to mature aerobic granular sludge; this species constituted about 0.5 ± 0.1% of the biomass at the end of experiment. A survey of activated sludge WWTPs found that the most numerous bacterial genera were *Tetrasphaera*, *Trichococcus*, *Candidatus Microthrix*, *Rhodoferax*, *Rhodobacter*, and *Hyphomicrobium*, p-55-a5 belonging to Firmicutes and P2CN44 and B45 belonging to the phyla Chloroflexi (McIllroy et al. 2015), while commonly occurring genera were *Zoogloea*, *Dechloromonas*, *Prosthecobacter*, *Caldilinea*, and *Tricoccus* (Zhang et al. 2012). The results of the present study show that, out of the abovementioned bacterial genera, only *Tetrasphaera*, *Dechloromonas*, and *Zoogloea* were common in the mature granular sludge in the full-scale facility; the other genera were not present in large numbers. However, the abundance of *Ohtaekwangia* (Cytophagia), *Sphingopyxis* (Sphingomonadales), and *Flavobacterium* (Flavobacteria) was much higher than what has been reported in activated sludge systems, indicating that these genera may be involved in granulation as structural microorganisms. Both *Flavobacterium* sp. and *Sphingopyxis* sp. have been previously identified in anaerobic granular sludge reactors (Kim et al. 2005; Aslam et al. 2005). *Sphingopyxis* sp. are aerobic, methylotrophic bacteria that are able to grow using nitrate as a terminal electron acceptor and to accumulate poly-b-hydroxyalkanoates (Tobella et al. 2005; García-Romero et al. 2016), which might confer an environmental advantage in low-loaded aerobic granular sludge operated in anaerobic/oxic conditions. In the case of *Flavobacterium* sp., there are contradictory reports in the literature. In laboratory studies, *Flavobacterium* sp. was previously reported to make no contribution towards aerobic granular sludge formation or to occur only during the granulation phases but not in mature aerobic granules (Adav et al. 2010b; Abdullah et al. 2013). On the other hand, *Flavobacterium* sp. was the predominant microbial genus in laboratory aerobic granular sludge reactors fed with glucose-based synthetic wastewater or in chloroaniline-degrading aerobic granules (Zhu et al. 2008; Li et al. 2008). Our results suggest that, in a full-scale facility, this genus was an important component of mature granules that supported both denitrification and granulation by production of EPS.

## Conclusions

Application of AGS allowed for more compact wastewater treatment than with activated sludge; after upgrading, the volume of the reactors was reduced by 30%, but the quality of the effluent was improved. AGS was easily thickened and dewatered, which allowed both the volumes of the tanks in the sludge treatment line and the volume of excess sludge to be decreased. The microorganisms responsible for creating the granule structure and removing nutrients in the full-scale facility were identified. After upgrading, microbial structure shifted from filamentous bacteria (mostly Actinobacteria) forming a backbone for activated sludge flocs to EPS-producing bacteria in aerobic granules (Sphingomonadales and Xanthomonadales). The abundances of Betaproteobacteria, Deltaproteobacteria, Flavobacteria, and Cytophagia, largely responsible for organic and nutrient removal, increased more than three times in biomass after upgrading, which significantly increased the treatment efficiency. These results are useful for designing WWTPs that use AGS or for upgrading existing facilities to AGS technology.

## Electronic supplementary material

ESM 1 (DOCX 34 kb)
