# Supplementary material for: Performance and microbial characteristics of biomass in a full-scale aerobic granular sludge wastewater treatment plant
Source: Environ Sci Pollut Res Int. 2017 Nov 3;25(2):1655–69. doi: 10.1007/s11356-017-0615-9 (PMC5766719; doi:10.1007/s11356-017-0615-9)
Supplement: Supplementary file 1 — (DOCX 34 kb) [file 11356_2017_615_MOESM1_ESM.docx]

**Performance and microbial characteristics of biomass in a full-scale aerobic granular sludge wastewater treatment plant**

Piotr Świątczak, Agnieszka Cydzik-Kwiatkowska*

University of Warmia and Mazury in Olsztyn, Department of Environmental Biotechnology, 10-709 Olsztyn, Słoneczna 45 G

*Corresponding author: agnieszka.cydzik@uwm.edu.pl, tel. +48 89 5234194, fax +48 89 5234131

**Supplementary Materials**
